# Draining Large Pericardial Effusion in a Pulmonary Hypertension Patient: Between a Rock and a Hard Place

**DOI:** 10.1002/ccr3.70370

**Published:** 2025-03-27

**Authors:** Azhar Farooqui, Mohamed Alama, Ibrahim Antoun

**Affiliations:** ^1^ Department of Cardiology Kettering General Hospital Kettering UK; ^2^ Department of Cardiovascular Sciences Leicester UK

**Keywords:** pericardial drain, pericardial effusion, pericardiocentesis, pulmonary hypertension, systemic lupus erythematosus

## Abstract

Managing pericardial effusion in autoimmune diseases like systemic lupus erythematosus becomes particularly challenging when accompanied by pulmonary hypertension (PH). The risk of acute right ventricular overload and hemodynamic collapse makes pericardial drainage a high‐stakes decision. This case highlights the necessity of a multidisciplinary, individualized approach in high‐risk patients. It also underscores the effectiveness of immunosuppressive therapy (e.g., mycophenolate mofetil) and pulmonary vasodilators (e.g., Ambrisentan, Tadalafil) in achieving gradual resolution. Close collaboration with PH specialists is essential for safely navigating these complex cases.

**Summary**

- Pericardial effusion in autoimmune diseases such as systemic lupus erythematosus, especially in the presence of coexisting pulmonary hypertension (PH), poses a significant management challenge due to the high risk of acute right ventricular overload and haemodynamic collapse associated with drainage.
- This case highlights the importance of a multidisciplinary, individualised approach, refraining from immediate drainage in high‐risk patients, and underscores the efficacy of immunosuppressive therapy (e.g., mycophenolate mofetil) and pulmonary vasodilators (e.g., Ambrisentan, Tadalafil) for gradual resolution.
- Collaborative care with PH specialists is crucial to safely managing these complex scenarios.

## 1 Introduction

Pericardial effusion, characterized by abnormal fluid accumulation within the pericardial sac, is a known complication in connective tissue diseases such as systemic lupus erythematosus (SLE). When it occurs in conjunction with pulmonary hypertension (PH), it presents a unique and significant clinical dilemma. Pericardial effusions in PH patients increase the risk of cardiac tamponade and right ventricular (RV) failure. However, managing such effusions—particularly through drainage—carries considerable risk due to the complex hemodynamic changes associated with PH [1]. In particular, abrupt removal of the pericardial fluid in PH patients can precipitate acute RV dilation due to a sudden increase in venous return. This dilation, exacerbated by ventricular interdependence, may lead to compromised left ventricular (LV) filling and potential circulatory collapse [2].

In recent years, the literature has stressed the risks of pericardial drainage in PH patients, advocating for a more cautious approach. When drainage is clinically necessary, staged or low‐volume drainage over several days has been proposed as a safer alternative. Individualized decision‐making and the need for a multidisciplinary approach are particularly important in patients where pericardial drainage could exacerbate RV dysfunction and increase morbidity and mortality. We describe a case of a young female with PH secondary to SLE and significant pericardial effusion, along with the challenges in the decision‐making regarding drainage.

## 2 Clinical History and Examination

Our patient is a 27‐year‐old female who presented to our hospital with symptoms of breathlessness. The symptoms have progressively worsened over the last 2 months, associated with a dry cough. She had a background of Sjogren's syndrome, systemic lupus erythematosus (SLE) and asthma. She had never received immunosuppression as she had been otherwise asymptomatic under follow‐up by a local Rheumatologist. Her body mass index (BMI) was 28 kg/m^2^; she was a non–smoker with a negligible alcohol intake history. Her regular medications included inhalers for her asthma and combined oral contraceptive pills.

**FIGURE 1:**
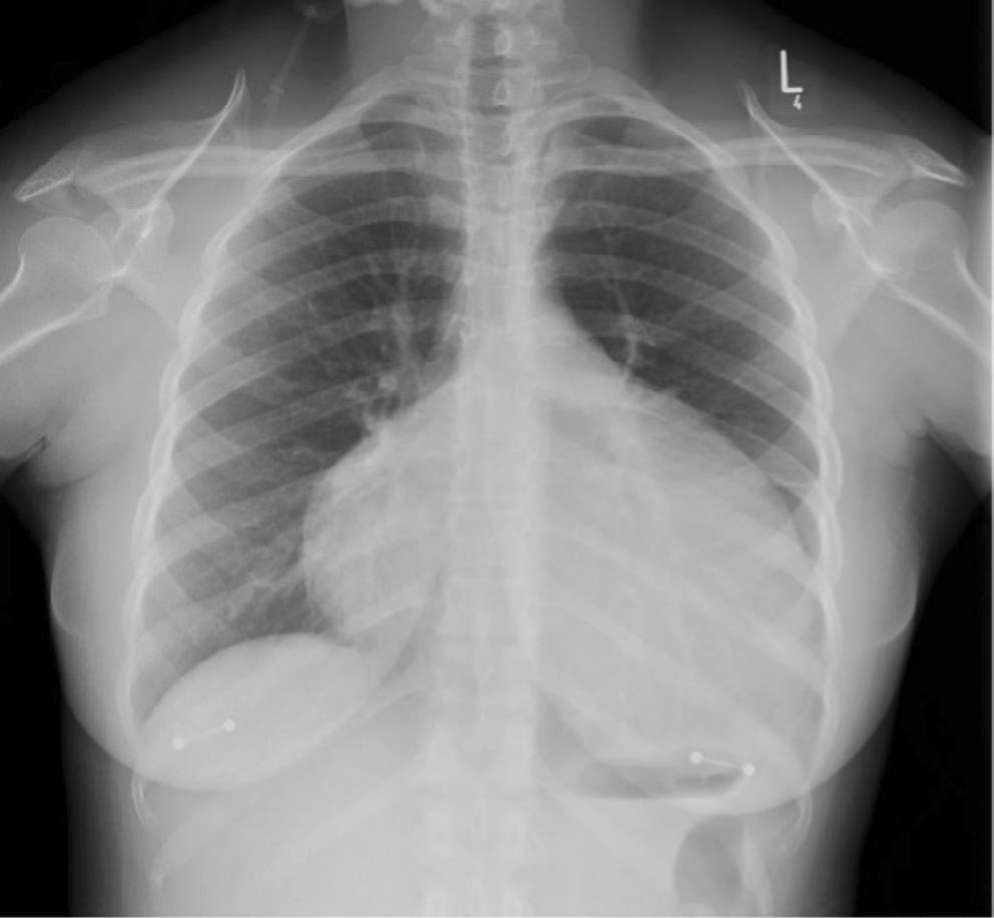
Chest X‐ray demonstrating a “globular shaped heart” should raise suspicion of pericardial effusion. Other differentials include an underlying significantly enlarged heart (cardiomegaly), such as in cases of dilated cardiomyopathy.

For her symptoms of breathlessness, she was previously seen by her general practitioner (GP) and was prescribed antibiotics (Amoxicillin and then doxycycline) for presumed respiratory tract infections. Unfortunately, this did not help, and she was subsequently prescribed a 1‐week course of prednisolone that appeared to have helped her symptoms. Meanwhile, the GP arranged a chest X‐ray (CXR) and routine blood tests. Following an abnormal CXR (Figure 1), the GP referred the patient to medical same‐day emergency care for urgent review by the medical team.

At the hospital presentation, she was tachycardic with a heart rate of 112 beats per min (bpm), but all other observations were within normalranges.

## 3 Differential Diagnosis and Diagnostic Tests

**FIGURE 2:**
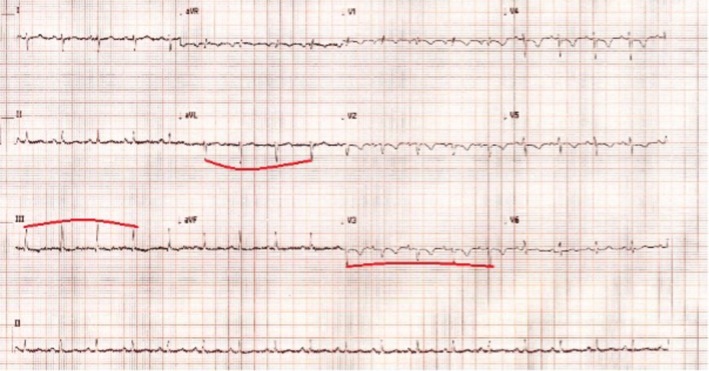
12 lead ECG demonstrates sinus rhythm with anterior T‐wave inversions. There is also evidence of alternating QRS complex amplitude (electrical alternans demonstrated via traced line), which is typically associated with pericardial effusion due to the swinging of the heart within the pericardial fluid‐filled space.

**FIGURE 3:**
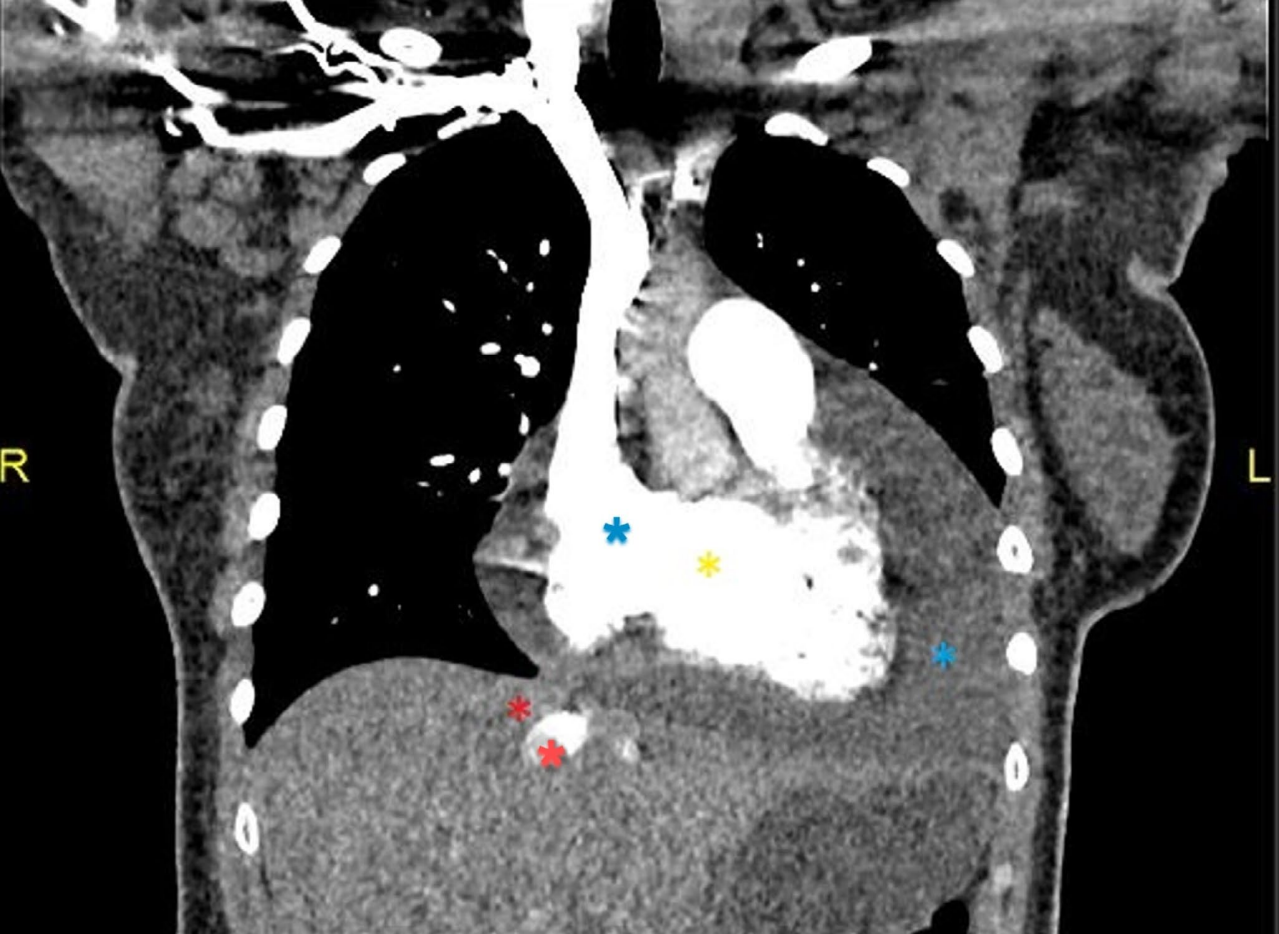
Computed tomography pulmonary angiogram demonstrates a marked pericardial effusion (yellow Asterix) with a dilated right ventricle (blue Asterix) and reflux of contrast into the hepatic veins denoting right‐sided cardiac failure (red Asterix).

**FIGURE 4:**
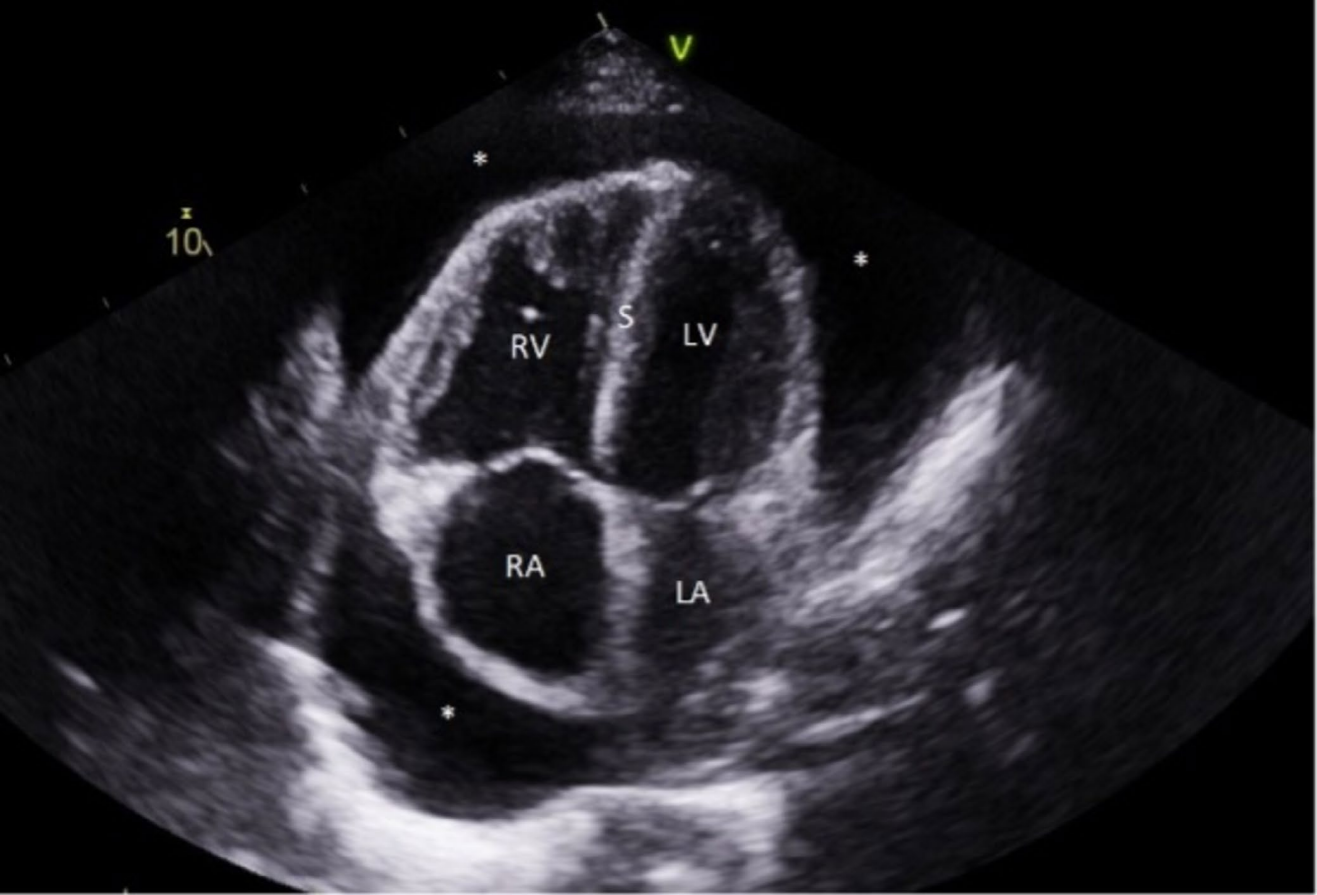
This 4‐chamber echocardiography view demonstrates a large pericardial effusion (*), with a dilated right ventricle and septal flattening (“S”) throughout the cardiac cycle, suggesting right ventricular pressure and volume overload. It also demonstrates a smaller left ventricular cavity. LA, left atrium; LV, left ventricle; RA, right atrium; RV, right ventricle; S, septum.

Blood investigations demonstrated that full blood count, electrolytes, kidney, and liver functions were insignificant. The 12‐lead electrocardiogram (ECG) is attached (Figure 2). She had an elevated D‐Dimer of 3355 ng/mL (reference < 500 ng/mL), necessitating a computed tomography pulmonary angiogram (CTPA), which ruled out a pulmonary embolism (PE) but showed large pericardial effusion, with a dilated right ventricle and reflux of contrast into the hepatic veins denoting right ventricular (RV) failure (Figure 3). It prompted the need for an urgent echocardiogram (Figure 4).

The detailed echocardiogram confirmed a large effusion without apparent tamponade physiology. It also showed significantly elevated estimated pulmonary artery systolic pressures, raising suspicion of pulmonary hypertension. Given the underlying symptoms, the dilemma was whether the pericardial effusion required urgent drainage. The pulmonary hypertension services at a specialized quaternary centre were contacted and advised against draining the effusion, as drainage in this subgroup has been associated with significant morbidity and mortality. Draining the pericardial effusion in patients with underlying pulmonary hypertension can cause a sudden increase in venous return, where acute right ventricular dilatation may compromise left ventricular filling (due to ventricular interdependence) and thus lead to circulatory collapse. Low‐volume effusion drainage over several days has been described in the literature as a way to reduce the risk of right ventricular dysfunction.

## 4 Results and Conclusion

This patient underwent an urgent transfer to the tertiary centre, where, after appropriate investigations, a formal diagnosis of pulmonary hypertension secondary to SLE was made using a right heart catheter. She was started on immunosuppression for SLE with mycophenolate mofetil and started on Ambrisentan (Endothelin‐1 receptor antagonist) and Tadalafil for pulmonary hypertension. Follow‐up echocardiograms 2 weeks later demonstrated gradual resolution of the pericardial effusion, with a scan 8 months later demonstrating a marked reduction compared to the initial diagnosis with symptomatic improvement. The patient remains under follow‐up by the pulmonary hypertension team. This case highlights the necessity of a multidisciplinary, individualized approach in high‐risk patients. It also underscores the effectiveness of immunosuppressive therapy (e.g., mycophenolate mofetil) and pulmonary vasodilators (e.g., Ambrisentan, Tadalafil) in achieving gradual resolution.

## 5 Discussion

This case highlights the unique challenges in managing pericardial effusion in patients with PH, particularly when associated with autoimmune diseases such as SLE [3]. Pericardial effusion in PH carries a high risk for adverse outcomes due to its potential to increase RV workload, ultimately leading to RV failure [4]. The physiological complexities in these patients are due to the interplay between pulmonary vascular resistance, RV function, and pericardial pressure dynamics [5]. In cases of PH, pericardial effusion may contribute to tamponade physiology, yet the traditional intervention of pericardial drainage is not straightforward. In PH patients, rapid decompression of the pericardial space can abruptly increase venous return, precipitating RV dilation and potentially compromising LV filling due to ventricular interdependence [2]. Such hemodynamic shifts risk causing circulatory collapse and have been associated with substantial morbidity and mortality in this patient group. Moreover, the timing and method of drainage are critical. In cases where pericardial effusion is associated with significant hemodynamic compromise, surgical intervention may be warranted [6]. Surgical options, such as pericardial window or pericardiotomy, may provide a more definitive solution, especially in patients with recurrent effusions or those who do not respond to percutaneous drainage [7]. However, these procedures carry their own risks and should be considered carefully in the context of the patient's overall clinical picture.

Given these risks, emerging literature supports a more conservative, individualized approach to managing pericardial effusions in PH patients [1, 8], reserving urgent pericardial drainage for instances of life‐threatening tamponade. Staged or low‐volume drainage over several days has been proposed as a safer alternative to sudden decompression, though evidence supporting this approach is largely observational. Vallabhajosyula and colleagues [9] shared a case of idiopathic PH, complicated with a large pericardial effusion and associated hemodynamic compromise where gradual drainage of 2.3 L over 15 days resulted in improvement in patient hemodynamics. Singh et al. [10] shared a case where their patient underwent serial, low‐volume pericardiocentesis over several days alongside real‐time invasive hemodynamic monitoring to detect signs of RV failure due to drainage.

Our case underscores the importance of identifying and managing the underlying aetiologies of PH. Prompt immunosuppression for SLE and the addition of PH‐specific therapies resulted in clinical improvement and effusion resolution. This reinforces that a multidisciplinary, tailored approach is paramount in managing high‐risk complications in autoimmune‐related PH.

## Author Contributions

**Azhar Farooqui:** conceptualization, data curation, writing – original draft. **Mohamed Alama:** writing – review and editing. **Ibrahim Antoun:** supervision, writing – review and editing.

## Consent

The authors confirm that written consent was obtained from the patient before submission of the case report.

## Conflicts of Interest

The authors declare no conflicts of interest.

## Data Availability

Data is available on request from the authors.

## References

[1] S. Sahay and A. R. Tonelli , “Pericardial Effusion in Pulmonary Arterial Hypertension,” Pulmonary Circulation 3, no. 3 (2013): 467–477. 24618534 10.1086/674302 PMC4070800

[2] R. A. Aqel , W. Aljaroudi , F. G. Hage , J. Tallaj , B. Rayburn , and N. C. Nanda , “Left Ventricular Collapse Secondary to Pericardial Effusion Treated With Pericardicentesis and Percutaneous Pericardiotomy in Severe Pulmonary Hypertension,” Echocardiography 25, no. 6 (2008): 658–661. 18652010 10.1111/j.1540-8175.2008.00661.x

[3] K. Parperis , N. Velidakis , E. Khattab , E. Gkougkoudi , and N. P. Kadoglou , “Systemic Lupus Erythematosus and Pulmonary Hypertension,” International Journal of Molecular Sciences 24, no. 6 (2023): 5085. 36982160 10.3390/ijms24065085 PMC10049584

[4] A. L. Hinderliter , P. W. Willis , W. Long , et al., “Frequency and Prognostic Significance of Pericardial Effusion in Primary Pulmonary Hypertension,” American Journal of Cardiology 84, no. 4 (1999): 481–484. 10468096 10.1016/s0002-9149(99)00342-2

[5] E. R. Fenstad , R. J. Le , L. J. Sinak , et al., “Pericardial Effusions in Pulmonary Arterial Hypertension: Characteristics, Prognosis, and Role of Drainage,” Chest 144, no. 5 (2013): 1530–1538. 23949692 10.1378/chest.12-3033

[6] O. Mirza , C. Patel , T. Shah , E. A. Hardin , S. Bartolome , and K. Chin , “Prognosis of Pulmonary Arterial Hypertension Patients With Pericardial Effusion Before and After Initiation of Parenteral Prostacyclin Therapy,” Pulmonary Circulation 13, no. 2 (2023): e12226, 10.1002/pul2.12226. 37063747 PMC10103584

[7] T. Mars , H. Mikolavčič , B. Salobir , and M. Podbregar , “Echocardiography of Isolated Subacute Left Heart Tamponade in a Patient With Cor Pulmonale and Circumferential Pericardial Effusion,” Cardiovascular Ultrasound 8, no. 1 (2010): 27. 20630052 10.1186/1476-7120-8-27 PMC2913935

[8] G. R. Honeycutt and Z. Safdar , “Pulmonary Hypertension Complicated by Pericardial Effusion: A Single Center Experience,” Therapeutic Advances in Respiratory Disease 7, no. 3 (2013): 151–159. 23258502 10.1177/1753465812471416 PMC3677714

[9] S. Vallabhajosyula and P. R. Sundaragiri , “Atypical Cardiac Tamponade in Severe Pulmonary Hypertension,” BML Case Reports 2015 (2015): bcr2014209187. 10.1136/bcr-2014-209187 PMC4368944 25777489

[10] A. Singh , R. Mosarla , K. Carroll , et al., “Pericardiocentesis in Severe Pulmonary Arterial Hypertension Guided by a Pulmonary Artery Catheter,” JACC: Case Reports—Journal of the American College of Cardiology 29 (2024): 12. 10.1016/j.jaccas.2024.102339 PMC11232420 38984206

